# Myeloma Cells Deplete Bone Marrow Glutamine and Inhibit Osteoblast Differentiation Limiting Asparagine Availability

**DOI:** 10.3390/cancers12113267

**Published:** 2020-11-05

**Authors:** Martina Chiu, Denise Toscani, Valentina Marchica, Giuseppe Taurino, Federica Costa, Massimiliano G. Bianchi, Roberta Andreoli, Valentina Franceschi, Paola Storti, Jessica Burroughs-Garcia, Rosa Alba Eufemiese, Benedetta Dalla Palma, Nicoletta Campanini, Eugenia Martella, Cristina Mancini, Jixiu Shan, Michael S. Kilberg, Giovanna D’Amico, Erica Dander, Luca Agnelli, Giancarlo Pruneri, Gaetano Donofrio, Ovidio Bussolati, Nicola Giuliani

**Affiliations:** 1 Department of Medicine and Surgery, University of Parma, 43126 Parma, Italy; martina.chiu@unipr.it (M.C.); denise.toscani@unipr.it (D.T.); valentina.marchica@unipr.it (V.M.); giuseppe.taurino@unipr.it (G.T.); federica.costa@unipr.it (F.C.); massimiliano.bianchi@unipr.it (M.G.B.); roberta.andreoli@unipr.it (R.A.); paola.storti@unipr.it (P.S.); jib6x6@gmail.com (J.B.-G.); eufemieserosalba@gmail.com (R.A.E.); benedetta.dallapalma@gmail.com (B.D.P.); 2 Department of Medical-Veterinary Science, University of Parma, 43121 Parma, Italy; valentina.franceschi@unipr.it (V.F.); gaetano.donofrio@unipr.it (G.D.); 3 Hematology, Azienda Ospedaliero-Universitaria di Parma, 43126 Parma, Italy; 4 Pathological Anatomy, “Azienda Ospedaliero-Universitaria di Parma”, 43126 Parma, Italy; nicoletta.campanini@unipr.it (N.C.); emartella@ao.pr.it (E.M.); cmancini@ao.pr.it (C.M.); 5 Department of Biochemistry and Molecular Biology, University of Florida College of Medicine, Gainesville, FL 32610, USA; shanjx@ufl.edu (J.S.); mkilberg@ufl.edu (M.S.K.); 6 Centro Ricerca Tettamanti, Pediatric Department, University of Milano-Bicocca, Fondazione MBBM, 20900 Monza, Italy; giovanna.damico@asst-monza.it (G.D.); e.dander@asst-monza.it (E.D.); 7 Department of Pathology-Fondazione IRCCS Istituto Nazionale dei Tumori, 20133 Milano, Italy; luca.agnelli@unimi.it (L.A.); giancarlo.pruneri@istitutotumori.mi.it (G.P.)

**Keywords:** multiple myeloma, glutamine, osteoblast, bone disease, asparagine, glutaminase, SNAT2, asparagine synthetase, glutamine synthetase

## Abstract

**Simple Summary:**

Osteolytic bone lesions represent an important clinical feature of multiple myeloma (MM). MM cells metabolize very high amounts of glutamine (Gln) and significantly lower Gln in the bone marrow. In this contribution we demonstrate that MM-dependent Gln depletion impairs the differentiation of bone marrow mesenchymal stromal cells into osteoblasts, the cells that form new bone tissue. We also found that osteoblast differentiation is associated with increased expression of glutaminase, the main enzyme that metabolizes Gln, SNAT2, a transporter able to accumulate Gln into the cells, and asparagine synthetase, the enzyme that uses Gln to obtain asparagine (Asn). Asn rescued osteoblast differentiation of Gln-starved mesenchymal stromal cells. These results demonstrate that MM cells impair osteoblast differentiation, hindering mesenchymal Asn synthesis through Gln depletion. Besides providing a metabolic mechanism underlying osteolytic lesions in MM, these results suggest that Asn supplementation may prevent bone disease in MM patients.

**Abstract:**

Multiple myeloma (MM) cells consume huge amounts of glutamine and, as a consequence, the amino acid concentration is lower-than-normal in the bone marrow (BM) of MM patients. Here we show that MM-dependent glutamine depletion induces glutamine synthetase in stromal cells, as demonstrated in BM biopsies of MM patients, and reproduced in vitro by co-culturing human mesenchymal stromal cells (MSCs) with MM cells. Moreover, glutamine depletion hinders osteoblast differentiation of MSCs, which is also severely blunted by the spent, low-glutamine medium of MM cells, and rescued by glutamine restitution. Glutaminase and the concentrative glutamine transporter SNAT2 are induced during osteoblastogenesis in vivo and in vitro, and both needed for MSCs differentiation, pointing to enhanced the requirement for the amino acid. Osteoblastogenesis also triggers the induction of glutamine-dependent asparagine synthetase (ASNS), and, among non-essential amino acids, asparagine rescues differentiation of glutamine-starved MSCs, by restoring the transcriptional profiles of differentiating MSCs altered by glutamine starvation. Thus, reduced asparagine availability provides a mechanistic link between MM-dependent Gln depletion in BM and impairment of osteoblast differentiation. Inhibition of Gln metabolism in MM cells and supplementation of asparagine to stromal cells may, therefore, constitute novel approaches to prevent osteolytic lesions in MM.

## 1. Introduction

Metabolic alterations of cancer cells, aimed at sustaining their uncontrolled growth, can alter the biochemical features of the tumor microenvironment and affect the metabolic behavior of other cell populations. Different studies have demonstrated that multiple myeloma (MM) cells undergo several changes in metabolism [1,2]. In particular, our group has shown that malignant plasma cells (PCs) do not express glutamine synthetase (GS), are highly glutamine (Gln)-addicted, consume huge amounts of the amino acid to sustain anaplerosis through glutaminase (GLS), and exhibit increased Gln uptake through the ASCT2 transporter [3]. Consequently, the concentration of Gln in bone marrow (BM) plasma of MM patients was found lowered by 30% in comparison with patients with indolent monoclonal gammopathies as smoldering MM (SMM) or monoclonal gammopathy of uncertain significance (MGUS) [3], as also reported by others [4].

In MM, the BM microenvironment plays an important role in malignant PCs survival and proliferation [5,6]. Moreover, the interaction between MM cells and BM stroma is responsible for the pathophysiology of osteolytic bone lesions that are the hallmark of MM [7,8,9]. As far as osteoblasts (OBs) are concerned, their differentiation and activity are impaired in MM, with severe suppression of the bone formation process that is critical for the development of osteolytic lesions [10,11,12].

The role of MM-dependent alterations of Gln metabolism in the development of MM bone disease has been never investigated. However, previous works showed that the absence of Gln prevents OB differentiation to a mineralizing phenotype [13] and that age-related bone loss is characterized by decreased Gln consumption together with defective OB differentiation [14]. Importantly, Gln contributes to the energy production necessary for the active biosynthesis of the osteogenic matrix in OB precursors [15]. Moreover, in murine models, bone mass formation is sustained by Gln metabolism [16], the inhibition of which also decreases OB differentiation of human mesenchymal stromal cells (MSCs) [17].

Overall, these data suggest a possible involvement of defects of Gln metabolism in MM-induced osteolysis, but little information is available from the studies on which Gln-dependent pathway may be important for OB differentiation. Indeed, Gln is both a direct precursor of asparagine (Asn), through the operation of asparagine synthetase (ASNS), and of glutamate (Glu), through GLS; in turn, Glu provides carbon moieties for the synthesis of many other non-essential amino acids and of glutathione.

Based on this literature evidence, we investigate how Gln shortage may be involved in the pathogenesis of OB suppression in MM and identify Asn availability as a link between Gln and the osteogenic differentiation process.

## 2. Results

### 2.1. Gln Consumption by MM Cells Induces GS in MSCs and Inhibits Their OB Differentiation

**Figure 1:**
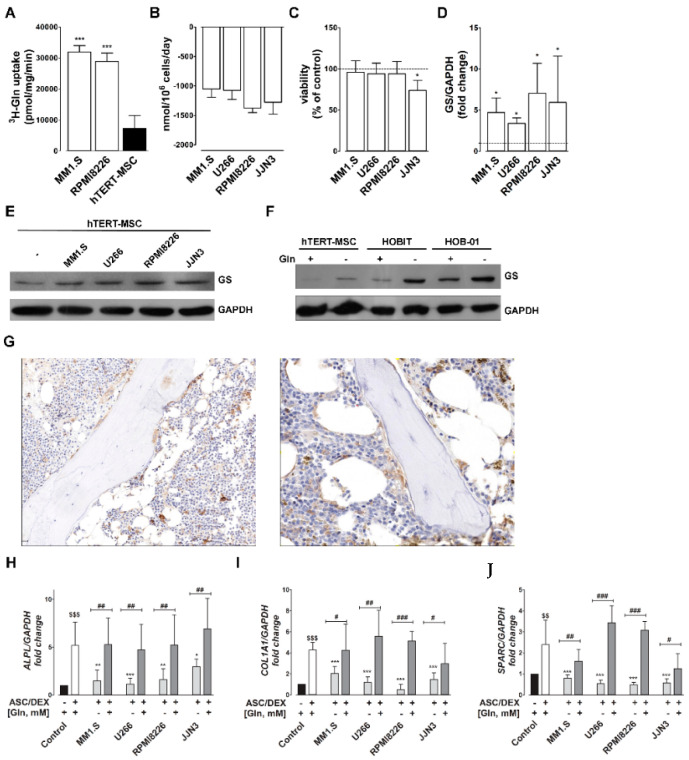
High Gln consumption by HMCLs causes GS induction in mesenchymal stromal cells (MSCs) and inhibits osteoblast (OB) differentiation. (**A**) 1-Min uptake of ^3^H-Gln (2 mM, 10 µCi/mL) was determined in MM1.S, RPMI8226 multiple myeloma (MM) cell lines and in hTERT-MSC cells. Data are presented as means ± SD of five independent determinations. *** *p* < 0.001 vs. hTERT-MSCs, as assessed with a two-tail Student’s *t* test for unpaired data. (**B**) Extracellular Gln consumption in MM cells. Data are presented as the difference between medium Gln concentration at t0 and after 3d of incubation, and are normalized on cell number and day of treatment. Means ± SD of three independent experiments are shown. (**C**) hTERT-MSCs were incubated in monoculture or in coculture with MM cells. After 72 h, cell viability was assessed and data were expressed as % of the value obtained with cells in monoculture. Data are presented as means ± SD of five independent determinations. * *p* < 0.05, as assessed with a two-tail Student’s *t* test for unpaired data. (**D**,**E**) Western blot of GS in hTERT-MSCs in monoculture (−) or in coculture with MM cells for 24 h. GAPDH was used for loading control. (**D**) Densitometry of GS expression in hTERT-MSCs kept in monocultures or cocultured with the indicated HMCLs. Data are expressed as fold changes relative to hTERT-MSCs in monoculture kept = 1. Means ± SD of three independent experiments are shown. * *p* < 0.05, as assessed with a one-sample *t* test. (**E**) A representative experiment is shown. (**F**) Representative Western blot of GS in hTERT-MSCs, HOBIT and HOB-01cells incubated for 24 h in the presence (+) or in the absence (−) of 2 mM Gln. GAPDH was used for loading control. (**G**) Examples of immunohistochemical staining of GS in bone marrow biopsies of 2 representative MM patients. (**H**–**J**) Expression of *ALPL* (**H**), *COL1A1* (**I**) and *SPARC* (**J**), in hTERT-MSCs incubated for 14 days under osteogenic conditions (ASC/DEX, +) in medium conditioned by HMCLs, supplemented with fresh Gln (+) or without Gln supplementation (−). Control cells were differentiated in fresh osteogenic medium at 2 mM Gln. Gene expression was normalized on *GAPDH* expression. Data are expressed as fold changes relative to undifferentiated hTERT-MSCs kept at 1. Means ± SD of three independent experiments are shown. $$ *p* < 0.1 and $$$ *p* < 0.01 vs. undifferentiated hTERT-MSCs as assessed with a one-sample *t* test. * *p* < 0.05, ** *p* < 0.01, and *** *p* < 0.001 vs. control differentiated hTERT-MSCs and # *p* < 0.05, ## *p* < 0.01, and ### *p* < 0.001 as assessed with a two-tail Student’s *t* test for unpaired data.

As expected [3], human myeloma cell lines (HMCLs) had a much higher transport of Gln than hTERT-MSCs (Figure 1A) and rapidly consumed a substantial portion of the extracellular Gln (Figure 1B). However, when co-cultured with HMCLs, MSCs viability was not significantly affected, with the exception of a modest decrease upon co-culture with JJN3 cells (Figure 1C). Consistently, Gln deprivation only moderately affected hTERT-MSCs, with an EC_50_ for Gln around 75 µM, but markedly hindered the viability of OB cell lines (EC_50_ for Gln around 275 µM, Supplementary Materials Figure S1).

Co-culture with any of the four HMCL tested caused a marked induction of GS in hTERT-MSCs (Figure 1D,E). GS was also induced upon Gln depletion in both hTERT-MSCs and OB cell lines (Figure 1F). As shown in Figure 1G, OBs, but not PCs, were strongly positive for GS in all the BM biopsies tested in a series of MM patients.

When hTERT-MSCs were incubated for 14 days under osteogenic conditions, a marked induction of OB markers was observed (Figure 1H–J). However, if the same osteogenic conditions were applied to hTERT-MSCs incubated with conditioned medium of HMCLs ([Gln] = 0.37 ± 0.02 mM), OB marker expression was significantly inhibited. When [Gln] was restored to the control value of 2 mM, a partial or complete rescue of every OB marker was observed, independently of the HMCL used for medium conditioning (Figure 1H–J).

### 2.2. Gln Deprivation Hinders the Expression of OB Markers in MSCs

**Figure 2:**
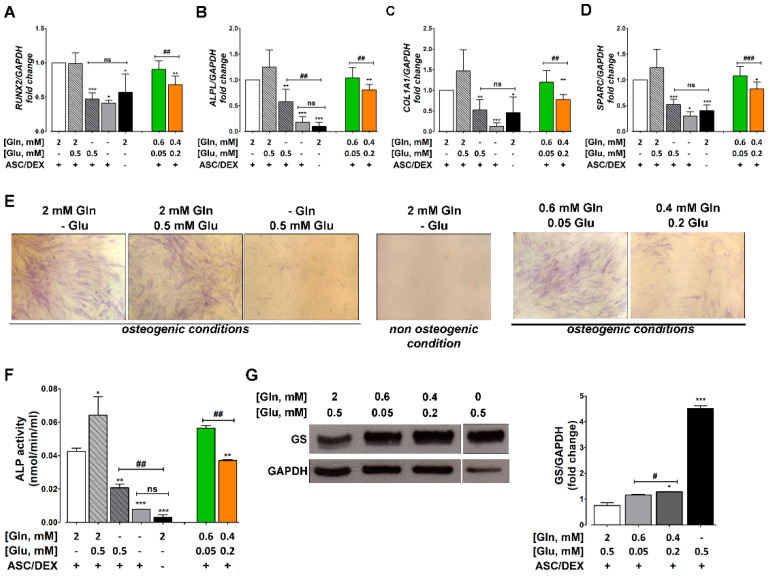
Gln depletion inhibits OB differentiation. (**A**–**D**) RT-PCR analysis of OB markers RUNX2 (**A**), ALPL (**B**), COL1A1 (**C**), and SPARC (**D**) in hTERT-MSCs incubated in osteogenic medium (ASC/DEX, +) at the indicated concentrations of Gln (mM) and Glu (mM) for 14 days. Gene expression was normalized on GAPDH expression. Data are expressed as fold changes relative to hTERT-MSCs differentiated at 2 mM Gln = 1. Means ± SD of three independent experiments are shown. * *p* < 0.05, ** *p* < 0.01, and *** *p* < 0.001 as assessed with one-sample *t* test. ## *p* < 0.01 and ### *p* < 0.001, as assessed with a two-tail Student’s *t* test for unpaired data. (**E**) ALP staining in hTERT-MSCs incubated in the presence or in the absence of osteogenic medium the indicated concentrations of Gln and Glu for 14 days. Original magnification 100×. (**F**) ALP activity in hTERT-MSCs incubated in standard (ASC/DEX, −) or osteogenic medium (ASC/DEX, +) with the indicated concentrations of Gln (mM) and Glu (mM) for 14 days. Means ± SD of three independent experiments are shown. * *p* < 0.05, ** *p* < 0.01, and *** *p* < 0.001 vs. hTERT-MSCs differentiated in the presence of 2 mM Gln, as assessed with a two-tail Student’s *t* test for unpaired data. ## *p* < 0.01 as assessed with a two-tail Student’s *t* test for unpaired data. (**G**) Left panel, Western blot of GS in hTERT-MSCs incubated in osteogenic medium with the indicated concentrations of Gln (mM) and Glu (mM) for 14 days. GAPDH was used for loading control. A representative experiment is shown. Right panel, densitometry analysis of GS expression. Means ± SD of three independent experiments are shown. * *p* < 0.05 and *** *p* < 0.001 vs. hTERT-MSCs differentiated in the presence of 2 mM Gln, as assessed with a two-tail Student’s *t* test for unpaired data. # *p* < 0.05 as assessed with a two-tail Student’s *t* test for unpaired data.

As shown in Figure 2, when incubated for 14 days in osteogenic medium, hTERT-MSCs expressed significantly lower levels of OB markers in the absence than in the presence of Gln (Figure 2A–D). The expression of OB markers obtained in a Gln-free osteogenic medium was comparable to that observed in undifferentiated cells. The addition of 0.5 mM Glu neither hindered the expression of OB markers in the presence of Gln nor rescued MSCs differentiation in the absence of Gln. A significant decrease of OB markers was also detected when the incubation in the osteogenic medium was performed at 0.4 mM Gln/0.2 mM Glu (the average amino acid levels measured in the BM plasma of MM patients (3)) compared with the physiological Gln and Glu concentrations of 0.6 and 0.05 mM, respectively. Under osteogenic conditions, ALP staining (Figure 2E) was much more evident at 2 mM of Gln than in the absence of the amino acid. Instead, no clear-cut difference was noted between cells incubated at 0.5 mM Glu or in its absence. Comparable results were obtained measuring ALP activity (Figure 2F). In contrast to OB markers, GS was progressively induced as the extracellular concentration of Gln was lowered (Figure 2E), while GS-silenced cells presented a higher OB marker expression than control cells (Figure S2).

### 2.3. OB Differentiation Is Associated with the Induction of the SNAT2 Transporter

**Figure 3:**
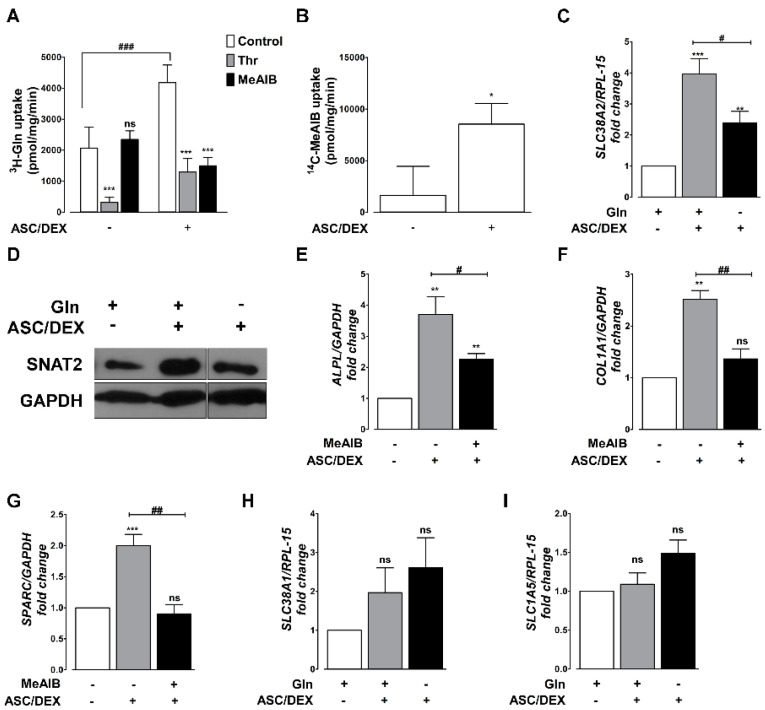
SNAT2 activity is required for osteoblastogenesis. hTERT-MSCs were incubated for 14 days in standard (ASC/DEX−) or osteogenic (ASC/DEX, +) medium. (**A**) 1-Min uptake of 3H-Gln (0.6 mM, 10 µCi/mL). Threonine (Thr) of 10 mM or 10 mM MeAIB were used to inhibit ASCT2 and SNAT2, respectively. *** *p* < 0.001 versus control, as assessed with a two-tail Student’s *t* test for unpaired data. ### *p* < 0.001 versus undifferentiated cells. (**B**) Sodium-dependent uptake of 1-min of 14C-MeAIB (0.1 mM, 2.5 µCi/mL). Data represent the sodium-dependent MeAIB uptake, obtained from the difference between uptake determined in the presence or in the absence of the cation * *p* < 0.001 versus control, as assessed with a two-tail Student’s *t* test for unpaired data. For A and B, data are means ± SD of three independent experiments with three independent determinations each. (**C**) RT-PCR analysis of SLC38A2 in hTERT-MSCs differentiated with osteogenic medium in the presence (+) or in the absence (−) of 2 mM Gln. Gene expressions were normalized on RPL-15 expression. Data are expressed as fold changes relative to undifferentiated cells maintained at 2 mM Gln = 1. Means ± SD of three independent experiments are shown. ** *p* < 0.01 and *** *p* < 0.001, as assessed with one sample *t* test. # *p* < 0.05, as assessed with a two-tail Student’s *t* test for unpaired data. (**D**) Western blot of SNAT2 in hTERT-MSCs undifferentiated (−) or differentiated (+) in the presence (+) or in the absence (−) of 2 mM Gln. A representative experiment is shown. (**E**–**G**) RT-PCR analysis of ALPL (**E**), and COL1A1 (**F**) and SPARC (**G**) in hTERT-MSCs differentiated with osteogenic medium in the presence (+) or in the absence (−) of 10 mM MeAIB. Gene expression was normalized on GAPDH expression. Data are expressed as fold changes relative to undifferentiated cells = 1. Means ± SD of three independent experiments are shown. ** *p* < 0.01 and ***p < 0.001 as assessed with a one sample *t* test. # *p* < 0.5 and ## *p* < 0.01, as assessed with a two-tail Student’s *t* test for unpaired data. (**H**,**I**) RT-PCR analysis of SLC38A1 (**H**) and SLC1A5 (**I**) in hTERT-MSCs differentiated with osteogenic medium in the presence (+) or in the absence (−) of 2 mM Gln. Gene expressions were normalized on RPL-15 expression. Data are expressed as fold changes relative to undifferentiated cells maintained at 2 mM Gln = 1. Means ± SD of three independent experiments are shown.

The data presented above suggest that OB differentiation depends on the availability of extracellular Gln and prompted us to assess the expression of Gln transporters. In mammalian cells, Gln transport is mostly sodium-dependent and attributable to the activity of SNAT transporters, coded by SLC38 genes, and of the ASCT2 transporter, encoded by SLC1A5. The contribution of these carriers was assessed determining Gln uptake in the presence of α-methylaminoisobutyric acid MeAIB, a specific substrate and competitive inhibitor of the SNAT transporters, or of L-threonine, a preferential substrate of ASCT2 [18]. The results indicated that Gln uptake doubled upon MSCs differentiation to OBs (Figure 3A). While no MeAIB-sensitive component of Gln transport was detectable in undifferentiated MSCs, more than 50% of Gln transport was inhibited by MeAIB in differentiated MSCs, pointing to a clear-cut induction of a SNAT transporter during osteoblastogenesis. This hypothesis was confirmed determining the sodium-dependent uptake of MeAIB (Figure 3B), which was almost undetectable in undifferentiated MSCs, but clearly sizable in differentiated cells. Differentiation was associated with a marked induction of SLC38A2, which encodes for the SNAT2 transporter, in the presence of Gln, while the induction was much smaller in the absence of the amino acid (Figure 3C,D). The addition of saturating concentrations of MeAIB produced a clear-cut decrease in the expression of ALPL, COL1A1, and SPARC (Figure 3E–G). The expression of SLC38A1, for the SNAT2-homologous SNAT1 transporter (Figure 3H), and that of SLC1A5 (Figure 3I), the gene for the ASCT2 transporter, were not significantly different in undifferentiated and differentiated MSCs. Conversely, glutaminase (GLS1, both KGA and GAC isoforms) exhibited a behavior comparable to SNAT2 (Figure S3A,B), and its inhibition hindered ALPL, COL1A1, and SPARC induction during MSCs differentiation (Figure S3C–E).

### 2.4. Gln-Related Gene Expression by Primary Human OBs and MSCs in MM Patients and HD

**Figure 4:**
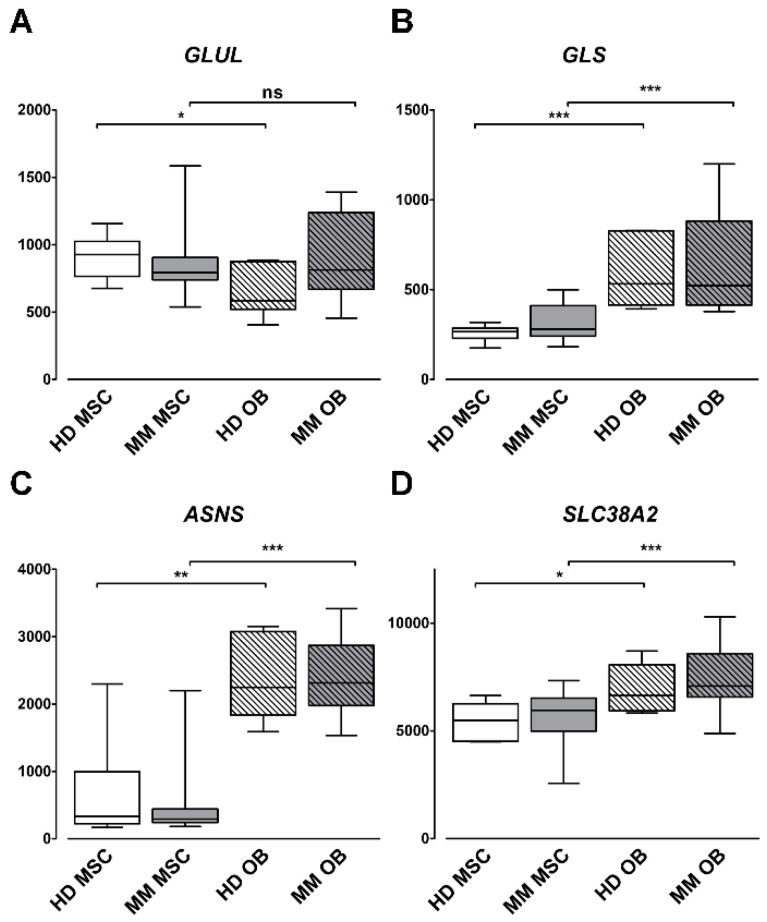
Primary human OBs express higher levels of ASNS, GLS, and SNAT2 than primary human MSCs. Box plot distribution of the expression levels of GLUL (**A**), GLS (**B**), ASNS (**C**), and SLC38A2 (**D**) in a dataset of 7 healthy donors (HDs) and 18 MM patients. * *p* < 0.05, ** *p* < 0.01, and *** *p* < 0.001, Kruskal–Wallis test.

The induction of Gln-related genes during MSCs differentiation led us to evaluate the transcriptional profile of the same genes in a proprietary dataset including undifferentiated primary human MSCs and primary human OBs, derived from BM biopsies of either healthy donors (HDs; *n* = 7) or MM patients (*n* = 16) [19]. The expression of GLUL, the gene for GS, was higher in undifferentiated MSCs than in OBs from HD, while, interestingly, no significant difference was detected in samples from MM patients (Figure 4A). On the other hand, SLC38A2 and GLS1, along with ASNS, the gene for Gln-dependent asparagine synthetase, showed a significantly increased expression in OBs compared to undifferentiated MSCs, in both HD and MM patients (Figure 4B–D). Eventually, no differences in MSCs from HD and MM were found, except for the SLC2A1 glucose transporter that was increased in MSCs from MM patients.

### 2.5. Asn Content Increases during OB Differentiation of MSCs

**Figure 5:**
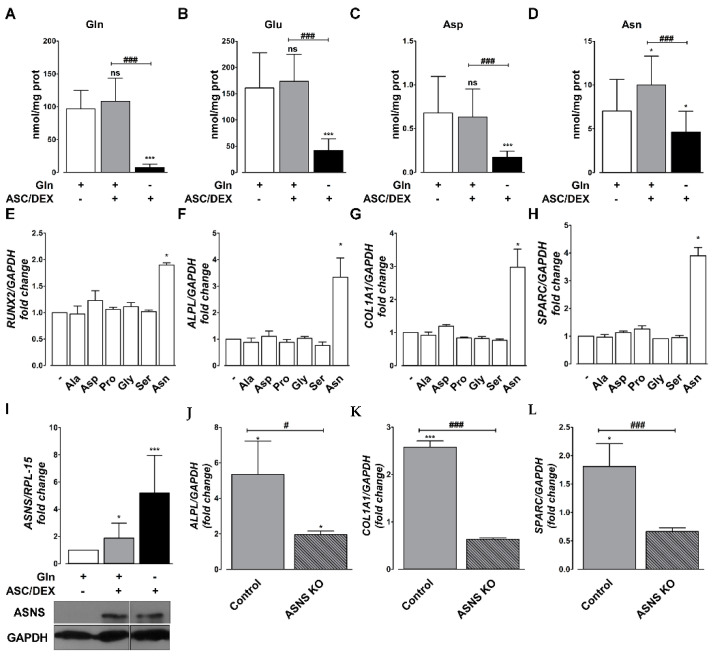
Asparagine is required for osteoblastogenesis. (**A**–**D**), The content of glutamine (**A**), glutamate (**B**), aspartate (**C**), and asparagine (**D**) in hTERT-MSCs incubated for 14 days in standard (−) or osteogenic medium (+) in the presence or absence of 2 mM Gln. Data are means of three independent determinations. * *p* < 0.05 and *** *p* < 0.001, vs. undifferentiated cells, ### *p* < 0.05, as assessed with a two-tail Student’s *t* test for unpaired data. (**E**–**H**), RT-PCR analysis of OB markers RUNX2 (**G**), ALPL (**H**), COL1A1 (**I**), and SPARC (**H**) in hTERT-MSCs incubated with osteogenic medium in the absence of Gln and in the presence of the indicated amino acids at the same concentration present in MEM. Gene expression was normalized on GAPDH expression. Data are fold changes relative to hTERT-MSCs differentiated in the absence of Gln, kept at 1 and are means ± SD of three independent experiments. * *p* < 0.05, as assessed with a one-sample *t* test. (**I**) RT-PCR analysis (upper panel) and Western blot (lower panel) of ASNS in hTERT-MSCs incubated for 14 days in standard (−) or osteogenic medium (+) in the presence or absence of 2 mM Gln. Gene expression was normalized on RPL-15 expression. Data are expressed as fold changes relative to undifferentiated cells with 2 mM Gln kept at 1. Means ± SD of three independent experiments are shown. * *p* < 0.05 and *** *p* < 0.001, as assessed with a one-sample *t* test. For Western blot, GAPDH was used as a loading control. A representative experiment is shown. (**J**–**L**) RT-PCR analysis of OB markers ALPL (**J**), COL1A1 (**K**), and SPARC (**L**) in hTERT-MSCs or ASNS KO-hTERT-MSCs incubated for 14 days in osteogenic medium in the presence of 2 mM Gln and 10 µM Asn. Gene expression was normalized on GAPDH expression. Data are expressed as fold changes relative to the undifferentiated control. Means ± SD of three independent experiments are shown. * *p* < 0.05 and *** *p* < 0.001, as assessed with a one-sample *t* test vs. undifferentiated control = 1. # *p* < 0.05 and ### *p* < 0.001, as assessed with a two-tail Student’s *t* test for unpaired data.

Subsequently, to characterize the metabolic mechanism underlying Gln effects on OB differentiation, we measured the cell content of Gln-related amino acids in differentiated MSCs. Gln content did not change appreciably after 14 days of incubation in the osteogenic medium but dramatically decreased upon Gln starvation (Figure 5A). A comparable behavior was exhibited by Glu (Figure 5B) and, with less evident changes, by aspartate (Figure 5C). On the contrary, cell Asn was higher in differentiated hTERT-MSCs than in undifferentiated cells, but the increase was abolished performing the incubation in the osteogenic medium in the absence of Gln (Figure 5D). Among the non-essential amino acids, only Asn was permissive for the induction of OB markers in hTERT-MSCs incubated in Gln-free osteogenic medium (Figure 5E–H). ASNS was induced, at both mRNA and protein levels (Figure 5I), in cells incubated under osteogenic conditions in the presence of Gln and, at much higher levels, in its absence. ASNS knock out (Figure S4) markedly blunted the induction of OB markers in hTERT-MSCs even in the presence of Gln (Figure 5J–L).

### 2.6. Asn Supplementation Corrects Changes in Transcriptional Profiles of hTERT-MSCs Induced by Gln Deprivation

The transcriptional profiles of hTERT-MSCs differentiated in the presence of Gln were compared with those obtained in cells differentiated in the absence of Gln or in the absence of Gln plus Asn. The analysis indicated that 274 genes were differentially expressed in cells differentiated with Gln compared with those differentiated in the absence of the amino acid. Moreover, 98 differentially expressed genes were found between cells cultured without Gln in the absence or in the presence of Asn; 74 of them were modulated by both Gln and Asn. Conversely, no gene was found differentially expressed between cells incubated with Gln and those without Gln but with Asn.

**Figure 6:**
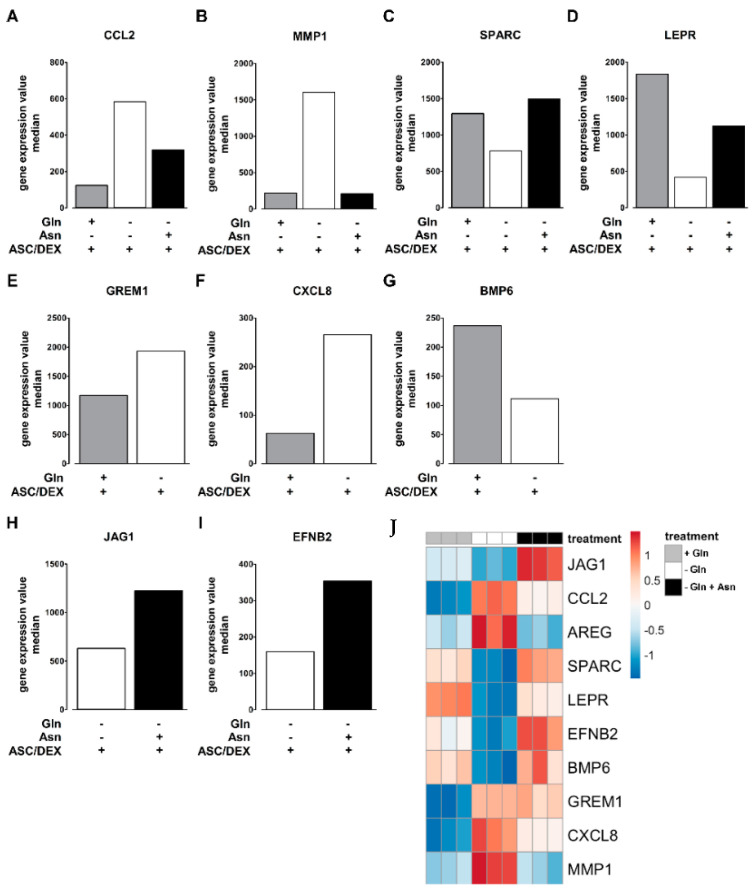
Transcriptional profiles of differentiating hTERT-MSCs: effects of Gln and Asn. hTERT-MSCs were differentiated in osteogenic media (ASC/DEX +) for 14 days with 2 mM Gln (+ Gln), without Gln (−Gln) or without Gln in the presence of 380 µM Asn (−Gln + Asn). (**A**–**I**) Bar graphs represent the median gene expression levels of selected genes significantly modulated by both Gln and Asn (**A**–**D**), exclusively by Gln (**E**–**G**) and Asn (**H**,**I**). (**J**) Heatmap of selected genes differently expressed in hTERT-MSCs differentiated with 2 mM Gln (+ Gln), without Gln (−Gln), and without Gln in the presence of 380 µM Asn (−Gln + Asn).

Some genes with a role in the relation with the BM microenvironment were further analyzed in detail. Specifically, AREG, CCL2, MMP1, GREM1, and CXCL8 were upregulated, whereas BMP6, SPARC, and LEPR were downregulated by Gln deprivation. Among them, CCL2, MMP1, SPARC, and LEPR were modulated by both Gln and Asn (Figure 6A–D), while GREM1, CXCL8, and BMP6 were exclusively modulated by Gln (Figure 6E–G). Conversely, JAG1 and EFNB2 were upregulated in the presence of Asn but not, or at least at lower levels, by Gln (Figure 6H,I). However, the heatmap of selected genes, reported in Figure 6J, indicates that, in most cases, the supplementation of Asn counteracts, or even overcompensates, the changes induced by Gln deprivation. These microarray data were further validated by real-time PCR (not shown).

### 2.7. Amino Acid Dependence of OB Differentiation of Primary BM MSCs

**Figure 7:**
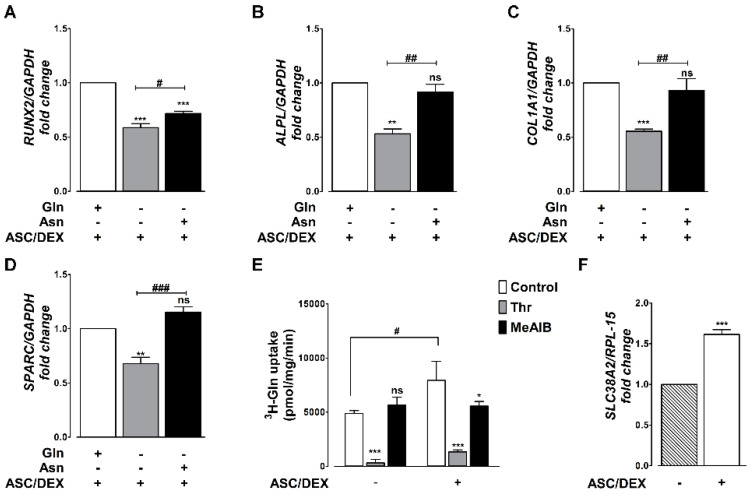
Primary MSCs require Gln or Asn for osteoblastogenesis. (**A**–**D**) RT-PCR analysis of *RUNX2* (**A**), *ALPL* (**B**), *COL1A1* (**C**), and *SPARC* (**D**) in primary MSCs from three different donors incubated in osteogenic medium (ASC/DEX, +) in the presence (+) or in the absence of 2 mM Gln or 380 µM Asn for 14 days. Gene expression was normalized on *GAPDH* expression. Data are expressed as fold changes relative to MSCs differentiated at 2 mM Gln = 1. Means ± SD of experiments performed with MSCs strains from different donors (*n* = 3) are shown. ** *p* < 0.01 and *** *p* < 0.001 as assessed with a one-sample *t* test. # *p* < 0.5, ## *p* < 0.01, and ### *p* < 0.001, as assessed with a two-tail Student’s *t* test for unpaired data. (**E**). Uptake of ^3^H-Gln of 1-min (0.6 mM, 10 µCi/mL) was performed in primary MSCs incubated for 14 days in standard (ASC/DEX, -) or osteogenic (ASC/DEX, +) medium. Threonine (Thr) of 10 mM or 10 mM MeAIB were used to inhibit ASCT2 and SNAT2, respectively. * *p* < 0.5 and *** *p* < 0.001 versus control, as assessed with a two-tail Student’s *t* test for unpaired data. # *p* < 0.001 versus undifferentiated cells. (**F**) RT-PCR analysis of *SLC38A2* primary MSCs incubated for 14 days in standard (ASC/DEX, −) or osteogenic (ASC/DEX, +) medium. Gene expression was normalized on *RPL-15* expression. Data are expressed as fold changes relative to undifferentiated cells maintained at 2 mM Gln = 1. Means ± SD of experiments performed with MSCs strains from three different donors are shown. *** *p* < 0.001 as assessed with a one-sample *t* test.

The role of Gln and Asn in OB differentiation was also assessed in primary MSCs obtained from the BM of three different HDs (Figure 7, Figure S5). Under osteogenic conditions, the same markers found upregulated in hTERT-MSCs were induced in primary BM MSCs (Figure S5). Moreover, Gln deprivation significantly hindered the induction of OB markers (Figure 7A–D), and Asn partially (for RUNX2, 7A) or fully complemented (for ALPL, COL1A1, and SPARC, Figure 7B–D) Gln deficiency. Lastly, as in hTERT-MSCs, Gln uptake significantly increased during OB differentiation, due to a sizable MeAIB-inhibitable fraction (Figure 7E), which was likely attributable to the induction of the SLC38A2/SNAT2 transporter (Figure 7F).

## 3. Discussions

Several interactions between MM cells and BM MSCs are responsible for the pathophysiology of bone disease [7,9,20]. The uncoupling between osteoclasts and OBs results in bone resorption and suppressed OB activity with a consequent decrease in bone formation [10,11]. However, bone disease has never been linked with the metabolic peculiarities of MM cells [1,2]. In particular, our group showed that MM cells were Gln addicted, used the amino acid for anaplerosis, had a negligible expression of GS, and, therefore, exclusively depended on the uptake of extracellular Gln to supply their craving for the amino acid. Consistently, MM patients, compared with subjects with SMM and MGUS, were characterized by higher BM levels of Glu associated with lower levels of Gln, a biochemical signature of enhanced glutaminolysis [3].

We demonstrated here that MM-induced Gln deprivation had several effects on BM MSCs. First, MSCs induced GS and, hence, exhibited a limited dependency on extracellular Gln, in contrast, the viability of OBs was markedly affected by Gln deprivation. Second, and more important, Gln depletion impaired OB differentiation of MSCs, with OB markers repressed upon the incubation with conditioned medium of MM cells, in which Gln concentration was markedly reduced, while OB differentiation of MSCs was rescued by the sole restoration of Gln levels. On the basis of these data, we hypothesized that MM cells create a peculiar low-Gln microenvironment that sustains GS expression in MSCs, affecting their behavior and differentiation potential. The histologic analysis of MM BM biopsies confirmed that, while malignant PCs are completely GS negative, as expected by previous results from our laboratory [3], a strong GS positivity is clearly detected in OBs, pointing to Gln shortage.

Our hypothesis was confirmed by the impairment of MSCs differentiation in the absence of Gln, a key finding confirmed in primary BM MSCs derived from three different healthy donors. The pivotal role for Gln in the regulation of OB differentiation is in line with previous works showing that the absence of Gln prevents OB differentiation to a mineralizing phenotype [13] and that age-related bone loss, associated with defective OB differentiation [14], involves decreased Gln metabolism. Moreover, recent contributions provided evidence that Gln contributes to energy production in OB precursors [15], and that skeletal stem cells metabolize Gln during OB differentiation, while GLS inhibition results in decreased bone mass in mice [16].

OB differentiation of MSCs was comparable at 0.5 mM Glu or in the absence of the anionic amino acid. This is apparently in contrast with literature data reporting Glu involvement in osteoblastogenesis. Indeed, Patton et al. [21] demonstrated that OBs express Glu metabotropic and ionotropic receptors, such as NMDA and AMPA, suggesting that they exploit Glu for autocrine and paracrine signaling. The role of Glu signaling may be only detectable when more complex culture systems are adopted, with the involvement of myeloid cells or osteoclast precursors.

Instead, OB differentiation was impaired even decreasing Gln from the physiological level of 0.6 to 0.4 mM, the average concentration detected in MM BM plasma. Thus, a relatively small decrease in Gln is sufficient to affect OB differentiation. These data pointed to the activity of a Gln sensor able to discriminate differences in the amino acid concentration in the millimolar range. We recently demonstrated that non-differentiated primary human MSCs do not express a sizable activity of SNAT transporters (K_m_ for Gln ranging between 1 and 2 mM) [22]. In agreement, we show here that the sodium-dependent uptake of the SNAT-specific substrate MeAIB was almost undetectable in undifferentiated MSCs. On the contrary, MeAIB uptake was clearly detected in differentiated hTERT-MSCs, and in differentiated primary MSCs, due to the induction of *SLC38A2,* which codes for the active transporter SNAT2 [23]. Interestingly, *SCL38A2* induction was blunted in the absence of Gln, suggesting that the induction of the transporter is an integral component of the chain of events leading to OB differentiation. The functional relevance of SNAT2-mediated Gln uptake for OB differentiation was demonstrated also by the failure to induce OB markers if MeAIB was present in the osteogenic medium at a concentration high enough to competitively suppress Gln uptake.

Several experiments were performed to identify the metabolic pathway involved in osteoblastogenesis. Gln metabolism seems necessary because GLS inhibition impaired the induction of OB markers (Figure S3). However, none of the Gln-related non-essential amino acids could replace the role of Gln in osteoblastogenesis except Asn. Consistently, the analysis of the cell content of Gln-related amino acids indicates that only Asn increased during OB differentiation, as long as Gln is available. *ASNS*, coding the Gln-dependent enzyme that synthesizes Asn from aspartate, was induced in the osteogenic medium in the presence and, even at higher levels, in the absence of Gln, when differentiation was blocked. To explain this apparent paradox, it should be recalled that *ASNS* regulation is extremely sensitive to the nutritional status of the cell [24]. Therefore, in the absence of Gln, ASNS was obviously upregulated, but in the absence of the obliged substrate Gln, it was ineffective and, consistently, Asn cell content markedly decreased, and differentiation was blocked. Differentiation was also blocked when *ASNS* was knocked out indicating that effective intracellular synthesis of Asn was required for successful differentiation. A similar dependence on ASNS expression and/or activity has also been described for endothelial differentiation [25,26].

In our cell model, Asn counteracted and in some cases overcompensated the transcriptional response to Gln depletion in MSCs, pointing to a role for the amino acid as a regulator of gene expression. However, since Asn is not catabolizable in mammalian cells [27], it is likely that the amino acid itself represents the metabolite required for OB differentiation. It is known that Asn is needed to maintain translation when extracellular Gln is scarce [28] and, interestingly, the synthesis of specific proteins seems particularly sensitive to Asn availability [29]. It is tempting to hypothesize that some proteins needed for osteoblastogenesis may be Asn-dependent. Moreover, Asn favors the uptake of serine and arginine through exchange transport systems thus promoting TORC1 activation [30]. Interestingly, TORC1 activity is needed in specific steps of OB differentiation [31].

The findings reported in this study were recounted in a model that connects defective osteoblastogenesis and partial depletion of BM Gln in MM. This model was supported also by the increased expression of *ASNS*, *SLC38A2,* and *GLS1* in primary human OBs compared with undifferentiated MSCs.

## 4. Materials and Methods

### 4.1. Patients

A histological retrospective analysis of bone biopsies was performed on a cohort of 15 patients with symptomatic MM (median age 64 years; 53% male, 47% female; International Staging System (ISS): I = 36% II = 29% III = 36%; median % plasma cells: 70, range %: 20–95) including 8 patients with newly diagnosed and 7 with relapsed MM, who had access to Hematology Unit of Parma. A total of 67% of MM patients carried type κ chain, 33% carried type λ chain, and 67% of MM patients had evidence of osteolytic lesions at the X-ray survey. Cylindrical iliac biopsies (3 mm in diameter, 10 mm in length) were obtained from the patients, fixed in sodium phosphate-buffered 4% paraformaldehyde at pH 7.4, dehydrated in a graded series of ethanol, and embedded in paraffin. Bone samples were longitudinally sectioned by means of a microtome cutting system (Leica S.p.A., Milan, Italy) to obtain 5-micron-thick sections, and underwent immunohistochemistry analysis.

### 4.2. Reagents, Cells, and Cell Culture Conditions

Reagents and cell lines used in the study were detailed in the supplemental data.

#### 4.2.1. Primary MSCs

Primary BM-derived MSCs were isolated by HDs by adherence to plastic dishes and then expanded in DMEM containing 10% FBS and supplemented with 1% penicillin–streptomycin. MSCs markers were confirmed using flow cytometry for the expression of CD90, CD73, CD105, and CD45. MSCs underwent 3 passages before being used in the assays presented herein.

#### 4.2.2. Co-Culture Experiments

For co-culture experiments, confluent, adherent hTERT-MSCs were cultured for 24 h or 72 h with JJN3, RPMI8226, MM1.S, and U266 HMCLs placed in the apical compartment of a trans-well insert (pore size 0.4 μM; Corning, Life Sciences, Amsterdam, The Netherlands).

Cell viability was assessed by adding resazurin (44 μM) to the incubation media [32]. After 2 h, fluorescence was measured at 572 nm with a fluorimeter (EnSpire^®^ Multimode Plate Readers, Perkin Elmer, Boston, MA, USA).

The conditioned medium was obtained culturing HMCLs for 24 h in DMEM supplemented with 5% FBS and 0.6 mM Gln, at a cell density of 250,000 cells/mL. At the end of the incubation, the medium was collected and centrifuged at 300 × *g* for 10 min, in order to remove MM cells and debris, and stored at −20 °C. Gln concentration in the medium was checked as described [3]. For the experiments, the medium was diluted 1:1 in DMEM supplemented with 5% FBS and 0.3 mM Gln (the average Gln concentration in HMCL conditioned medium) or 4 mM Gln (for Gln restoration).

#### 4.2.3. OB Differentiation Experiments

To induce the OB phenotype, both primary HD-MSCs and hTERT-MSCs were incubated for 14 days in the osteogenic medium, consisting of DMEM supplemented with ascorbic acid (ASC, 50 μg/mL), dexamethasone (Dex, 10^−8^ M), 5% FBS [33], or in standard growth medium in the presence of Gln or other non-essential amino acids, as indicated.

### 4.3. GLUL (Glutamine Synthetase) Knockdown in hTERT-MSCs

Lentivirus short hairpin RNA (shRNA) pool anti-*GLUL* (Origene, Rockville, MD, USA) was used for *GLUL* stable knockdown in hTERT-MSCs, with a scrambled sequence lentiviral vector used as a negative control. Recombinant lentivirus was produced by transient transfection of 293T cells following a standard protocol. hTERT-MSCs were infected and selected in culture with 400 ng/mL of puromycin. Selected clones were then screened for *GLUL* mRNA and/or protein expression. Stably transfected hTERT-MSCs were maintained in DMEM supplemented with 10% FBS and 400 ng/mL of puromycin until use.

### 4.4. ASNS Knockout in hTERT-MSCs

CRISPR-Cas9-mediated *ASNS* gene knockout was performed in hTERT-MSCs essentially by the protocol detailed by Cong et al. [34] and Ran et al. [35]. Two pairs of oligonucleotides encoding single strand guide RNAs (sgRNAs), which target 783 bp upstream (*ASNS*-KO-U) and 1106 bp downstream (*ASNS*-KO-D) relative to the transcription start site (TSS) of the human *ASNS* gene, were annealed and ligated into the vector pSpCas9(BB)-2A-Puro(PX459) V2.0 (Addgene, #62,988). For transfection, 2 × 10^6^ cells were plated in a 100-mm dish with 10 mL of RPMI1640 medium supplemented with 2 mM Gln, 1 µM hydrocortisone, 10% FBS, and antibiotics (streptomycin, penicillin G, and amphotericin B, ABAM, Gibco). After 24 h, cells were transfected with 5 µg each of plasmids containing *ASNS*-KO-U and *ASNS*-KO-D using lipofectamine 3000 following the manufacturer’s instructions (ThermoFisher, Waltham, MA, USA). After 1 h-incubation, cells were refilled with fresh RPMI1640 medium. After another 30 h, cells were passaged at a 1:10 dilution, and transfected cells were selected with 2 µg/mL of puromycin for 4 days. Cells were then cultured in growth medium for another 45–60 days until individual clonal colonies were evident. Genomic DNA from single clones was isolated with the Quick-DNA Microprep Kit (#D3021, Zymo Research, Irvine, CA, USA), and PCR with primers that amplified the junction of sgRNA-directed digestion was used to screen for those clones that contained a homozygous genomic deletion between *ASNS* nucleotides −783 to + 1106. The oligonucleotide sequences for the sgRNA at −783 were: *ASNS*-KO-U forward, 5′-AGCACATAATCATCTTGTGG-3′, and *ASNS*-KO-U reverse, CCACAAGATGATTATGTGCT-3′; whereas those for nt + 1106 were: *ASNS*-KO-D forward, 5′-AAGTGCTCAACAGTTACAGG-3′, and *ASNS*-KO-D reverse, 5′-CCTGTAACTGTTGAGCACTT-3′. PCR primers used for screening clones were: upstream junction forward, 5′-CAATGAACCTTAGAACAAGTCATCTCTC-3′, and reverse, 5′-GAAACACAGCGAGATCACATCTCTATA-3′; downstream junction forward, 5′-ATGATTCATAGCATTGCTGTAAGAGTAATT-3′, and reverse, 5′-ACTTCTAGAACAGTGCTGTCCAATAAATATA-3′.

### 4.5. Immunohistochemistry

Immunohistochemical staining of formalin fixed, paraffin-embedded tissue sections was performed on bone biopsies as described in the supplemental data.

### 4.6. Western Blot

Western blot analysis is detailed in the supplemental data.

### 4.7. Real Time-PCR Analysis

Real time-PCR analysis is detailed in the supplemental data.

### 4.8. AminoAacid Uptake

Gln and MeAIB uptake are detailed in the supplemental data.

### 4.9. Alkaline Phosphatase (ALP) Staining and Activity

ALP staining and activity are detailed in the supplemental data.

### 4.10. Liquid Chromatography Tandem Mass Spectrometry (LC–MS/MS)

Metabolite determination was performed as previously described [3]. Briefly, cells were seeded in a 24-well plate. After 14 days of differentiation, cells were washed with ice-cold PBS, and metabolites were extracted with 0.15 mL absolute ethanol. LC analyses were carried out with an Agilent HP 1100 pump coupled with a QTRAP 6500 + System (SCIEX).

### 4.11. Gene Expression Profiles Analysis

The transcriptional profiles of BM MSCs (7HD) and 16 MM patients) and OBs (7 HD and 16 MM patients) were generated as previously described [19]. The transcriptional profiles of hTERT-MSCs cultured under osteogenic conditions in the presence (2 mM) or absence of Gln or in the absence of Gln but in the presence of Asn (380 µM) were analyzed. To perform gene expression profiles, total RNA was purified using AllPrep DNA/RNA/miRNA isolation kit (Qiagen, Hilden, Germany), following the manufacturer’s protocol.

Preparation of biotin-labeled complementary RNA, hybridization to GeneChip ClariomD Arrays and scanning (GeneChip Scanner 3000 7G, Affymetrix Inc., Santa Clara, CA, USA) were performed according to manufacturer’s protocols.

The global expression profiles of 19,012 protein-coding and 13,972 long non-coding RNAs (lncRNAs) were extracted from GeneChip^®^ ClariomD arrays (Affymetrix, Thermo Fisher Scientific, USA), analyzed using RMA normalization procedure and annotations based on Gencode project (version 26) provided by the University of Michigan, as previously described [36]. The “samr” package was used in R for differential analyses. Data are publicly available on NCBI GEO repository under accession #GSE147791 (https://www.ncbi.nlm.nih.gov/geo/query/acc.cgi?acc=GSE147791).

### 4.12. Statistical Analysis

For statistical analysis, a two-tail Student’s *t* test for unpaired data was used, whenever not stated otherwise. GraphPad Prism 5.0™ was used for all the statistical analyses and *p* values < 0.05 were considered statistically significant.

### 4.13. Study Approval

This study was approved by the Institutional Ethical Review Board of Hospital of Parma, Italy, after informed consent according to the Declaration of Helsinki (approval n. 36895)

## 5. Conclusions

In conclusion, our study indicated that Gln-addicted MM cells lowered Gln in the BM microenvironment leading to a defective OB differentiation attributable, at least in part, to the impairment of Gln-dependent Asn synthesis. These results demonstrated for the first time the critical role of Gln-derived Asn shortage in MM-induced OB suppression and might provide novel strategies to treat MM osteolytic bone disease.

## Supplementary Materials

The following are available online at https://www.mdpi.com/2072-6694/12/11/3267/s1. Figure S1: OBs are more sensitive to extracellular Gln than MSCs, Figure S2: GS silencing enhances ALPL expression during MSCs differentiation, Figure S3: GLS activity is also needed for osteoblastogenesis, Figure S4: ASNS KO in hTERT-MSCs, Figure S5: Expression of OB markers during the differentiation of primary bone marrow MSCs. Figure S6: the whole western blots (uncropped blots).
