# Supplementary material for: Myeloma Cells Deplete Bone Marrow Glutamine and Inhibit Osteoblast Differentiation Limiting Asparagine Availability
Source: Cancers (Basel). 2020 Nov 5;12(11):3267. doi: 10.3390/cancers12113267 (PMC7694402; doi:10.3390/cancers12113267)
Supplement: Supplementary file 1 [file cancers-12-03267-s001.pdf]

cancers

MDPI

Supplementary Materials and Methods: Myeloma Cells Deplete Bone Marrow Glutamine and Inhibit Osteoblast Differentiation Limiting Asparagine Availability

Martina Chiu, Denise Toscani, Valentina Marchica, Giuseppe Taurino, Federica Costa, Massimiliano G. Bianchi, Roberta Andreoli, Valentina Franceschi, Paola Storti, Jessica Burroughs-Garcia, Rosa Alba Eufemiese, Benedetta Dalla Palma, Nicoletta Campanini, Eugenia Martella, Cristina Mancini, Jixiu Shan, Michael S. Kilberg, Giovanna D'Amico, Erica Dander, Luca Agnelli, Giancarlo Pruneri, Gaetano Donofrio, Ovidio Bussolati* and Nicola Giuliani*

Reagents, cells, and cell culture conditions

Serum was obtained from Lonza (Basel, Switzerland). Unless otherwise stated, Sigma Aldrich (Milan, Italy) was the source of all the other chemicals.

The human multiple myeloma–derived cell lines (HMCL) JJN3, RPMI8226, MM1.S and U266 were obtained from the European Collection of Cell Cultures and authenticated by DNA fingerprinting. The cells were periodically tested for *Mycoplasma* contamination, using the MycoProbe Mycoplasma Detection Kit purchased from R&D Systems. HMCL were cultured in Dulbecco's Modified Eagle's Medium (DMEM) low glucose (1g/l) supplemented with 2 mM glutamine (Gln), 10% fetal bovine serum (FBS) and antibiotics (100 U/ml penicillin, 100 μg/ml streptomycin). All cell lines were used between passages 4 and 15 from the time of purchase.

Immortalized human osteoblast-like (HOBIT) cells were kindly provided by Dr. B. L. Riggs (Rochester, MN, USA). The human pre-osteocytic cells (HOB-01) were established from human bone and kindly provided by Dr. Julia Billars (Collegeville, PA, USA). The human telomerase reverse transcriptase transduced mesenchymal stromal cell line (hTERT-MSCs) were kindly provided by Dr. D. Campana (S. Jude Hospital, Memphis, TN, USA) and cultured in DMEM low glucose supplemented with 2 mM Gln, 10% FBS and antibiotics (100 U/ml penicillin, 100 μg/ml streptomycin).

Amino acid uptake

Amino acid uptake was performed as described previously.[18] For the determination of α-methylaminoisobutyric acid (MeAIB) initial influx, cells were incubated for 90 min in EBSS (NaCl 117 mM, Tris-HCl 26 mM; KCl 5.3 mM, $CaCl_2$ 1.8 mM, $MgSO_4 \cdot 7H_2O$ 0.81 mM, choline phosphate 0.9 mM, glucose 5.5, supplemented with 0.02 % Phenol Red, adjusted at pH 7.4), rinsed with 200 μl of Na^+-free EBSS and incubated at pH 7.4 in EBSS or in a Na^+-free EBSS, both supplemented with α-[1-^{14}C]-MeAIB (2.5 μCi/ml, PerkinElmer). After 1 min, cells were washed with ice-cold urea (300 mM). Cell monolayers were extracted with 50 μl of cold absolute ethanol. The extracts were mixed with 200 μl of scintillation fluid and counted with a scintillation spectrometer (Microbeta^2, PerkinElmer, Milan, Italy). Data were expressed as pmol/mg protein/min.

Immunohistochemistry

Sections were stained with GS- Glutamine Synthetase primary antibody: (GS-6 Ventana-Roche; ready to use). After deparaffinization and rehydration, sections were treated with 3% hydrogen peroxidase for 5 mins. For antigen retrieval, sections were treated with pH9 Tris-EDTA buffer for 30 mins in water-bath at 98°C. The sections were immunostained with a polymeric system Ultraview 3,3′-diaminobenzidine DAB IHC Detection Kit (Ventana-Roche) in accordance with the manufacturer's specifications. DAB was used for staining development, and the sections were counterstained with hematoxylin. Negative controls consisted of substituting normal serum for the

primary antibody. Images were captured and analyzed by scanner Ventana DP200 (Ventana-Roche) to ensure the best image quality.

Western blot

Western blot was performed as previously described.[3] Blots were incubated at 4 °C overnight with the following antibodies: anti-GS (mouse, monoclonal, Catalog No. 610517, 1:1500, BD Transduction Laboratories, Franklin Lakes, NJ), anti-GAPDH (rabbit, polyclonal, Catalog No. G9545, 1:4000, Sigma-Aldrich), anti-SNAT2 (rabbit, polyclonal, Catalog No. Ab90677, 1:500, Abcam), anti-ASNS (mouse, polyconal, Catalog No. sc-365809, 1:2000, Santa Cruz).

ALP staining and activity

The ability of hTERT-MSC to differentiate into OBs was assessed using ALP staining and activity with an assay kit 14 days post-differentiation. Briefly, cells were fixed with cold formalin (4%) at 4 ℃, washed 3 times with 1X PBS, and stained for 30 minutes with the Alkaline Phosphatase semiquantitative histochemical kit (Sigma Aldrich) at room temperature, according to the manufacturer’s procedure. At the end, cells were washed three times with sterile water. Each condition was performed in duplicate.

ALP activity was assessed using a colorimetric Alkaline Phosphatase assay kit (Abcam, USA) according to manufacturer's protocol. ALP activity results were expressed as nmol/min/mL

Real time-PCR analysis

1 µg of total RNA, isolated with GeneJET RNA Purification Kit (Thermo Fisher Scientific, Waltham, MA USA), was reverse transcribed with RevertAid RT Reverse Transcription Kit (Thermo Fisher Scientific) following manufacturer’s instruction. For real time PCR (35 cycles), cDNA was amplified with PowerUp™ SYBR™ Green Master Mix (Applied Biosystem, Foster City, CA, USA) along with the following primers (5 pmol each): *RPL-15* (for 5′-GCAGCCATCAGGTAAGCCAAG-3′, rev 5′-AGCGGACCCTCAGAAGAAAGC-3′); SLC38A1 (for 5'-CACCACAGGGAAGTTCGTAATC-3', rev 5'-CGTACCAGGCTGAAAATGTCTC-3'); *SLC38A2* (for 5'-ATGAAGAAGGCCGAAATGGGA-3', rev 5'-TGCTTGGTGGGGTAGGAGTAG-3'); *SLC1A5* (for 5'-TGGTCTCCTGGATCATGTGG-3′, rev 5'-TTTGCGGGTGAAGAGGAAGT-3′); *ASNS* (for 5'-GATTGCCTTTCTGTTCAGTGTCT-3', rev 5'-GGGTCAACTACCGCCAACC-3'). Quantitative PCR was performed in a StepOneTM Real-Time PCR System (Applied Biosystems). Data analysis was made according to the Relative Standard Curve Method. The mRNA expression of *RUNX2* (hs01047973_m1), *COL1A1* (hs01076777_m1) *ALPL* (hs01029144_m1) and the two isoforms of GLS (*GAC* (hs01022166_m1), and *KGA* (hs01014019_m1)) was evaluated by Taqman® gene assay (Life Technologies, Thermo Fisher Scientific, Waltham, MA, USA). The mRNA expression of GADPH (assay ID: 141139) and SPARC (assay ID: 103218) was evaluated by Real-Time-ready Assays (Roche Diagnostics, Italy). Data were normalized to *RPL-15* or *GAPDH* expression.

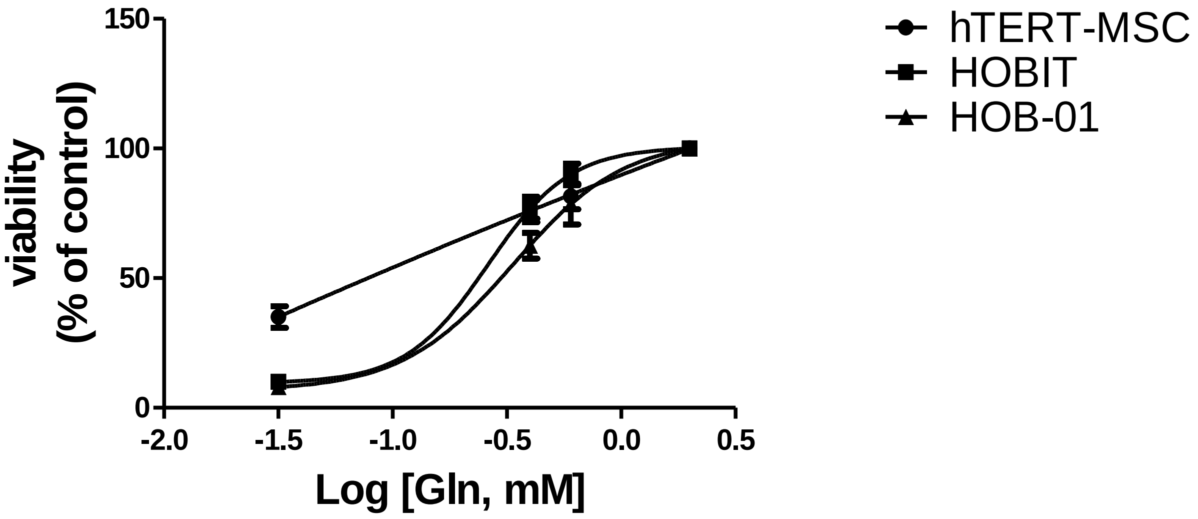

Figure S1. OBs are more sensitive to extracellular Gln than MSCs. hTERT-MSC, HOBIT and HOB-01 cell lines were incubated with increasing concentrations of extracellular Gln (0.03, 0.4, 0.6, 2 mM). After 72 h, cell viability was assessed, and data were expressed as % of the value obtained in cell maintained with 2 mM Gln. Data are presented as means ± SD of three experiment. Non-linear regression was obtained with GraphPad Prism™.

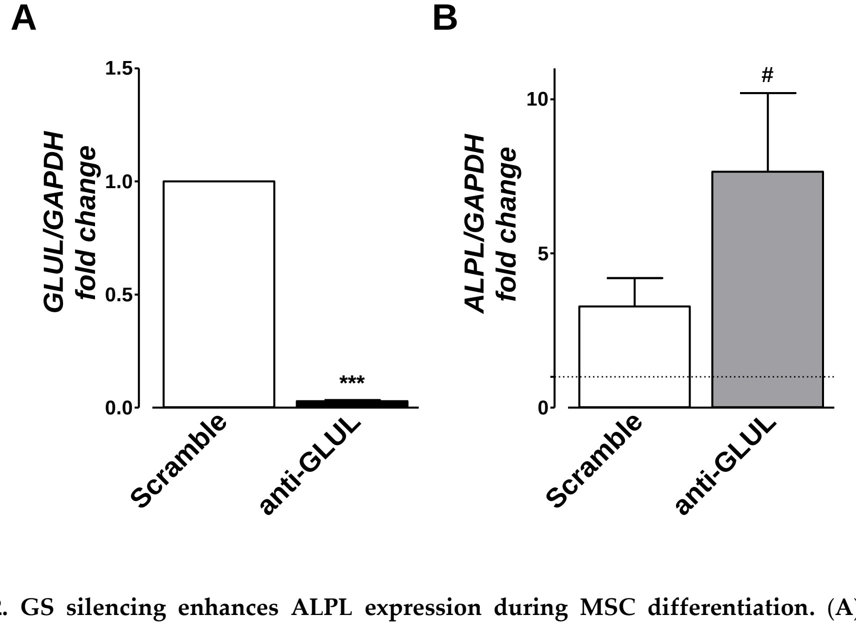

Figure S2. GS silencing enhances ALPL expression during MSC differentiation. (**A**) RT-PCR analysis of *GLUL* expression in scramble-transfected and *GLUL*-silenced hTERT-MSCs. Gene expression was normalized on *GAPDH* expression. Data are expressed as fold changes relative to scramble-transfected cells =1. Means ± SD of two independent experiments, each performed in duplicate, are shown. ****P*<.001 as assessed with one sample t test. (**B**) RT-PCR analysis of *ALPL* expression in scramble-transfected and *GLUL*-silenced hTERT-MSCs incubated in osteogenic medium. Gene expression was normalized on *GAPDH* expression. Data are expressed as fold changes relative to undifferentiated cells =1. Means ± SD of two independent experiments, each performed in duplicate, are shown. #P<0.5 as assessed with a two-tail Student's t test for unpaired data.

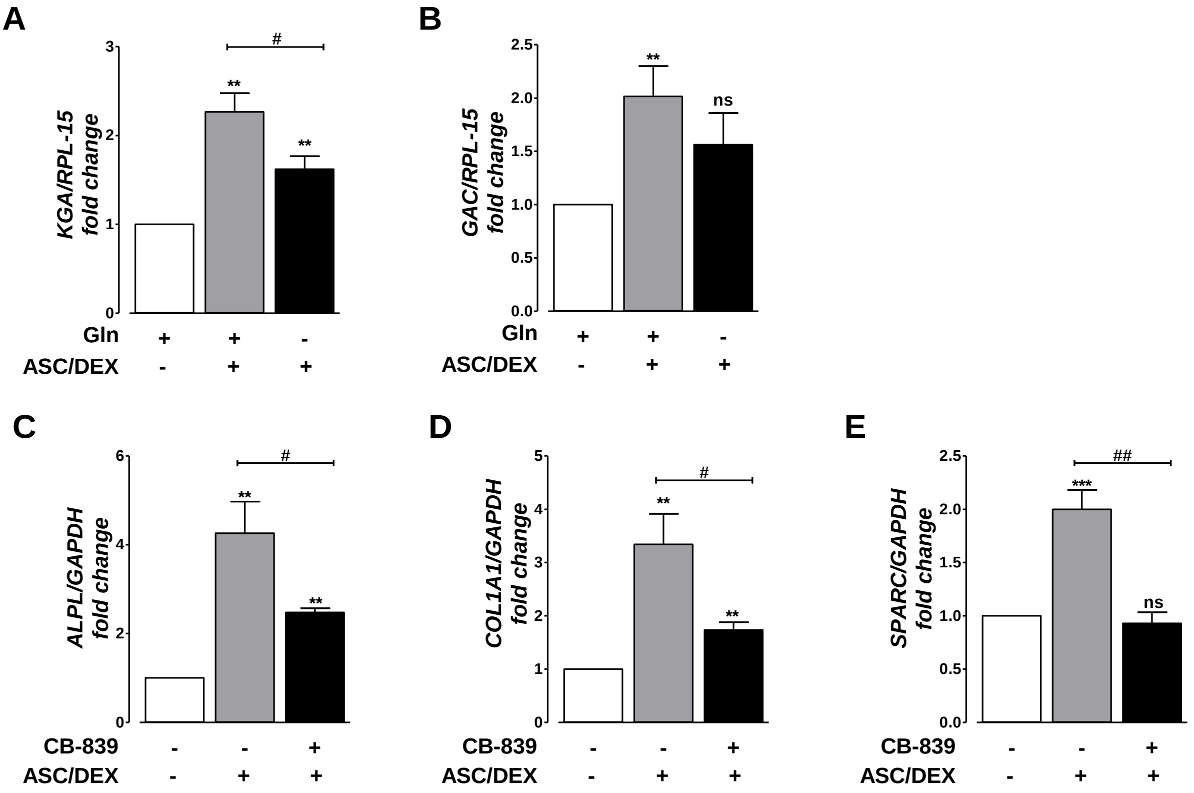

Figure S3. GLS activity is also needed for osteoblastogenesis. hTERT-MSCs were incubated for 14 days in standard (ASC/DEX, -) or osteogenic (ASC/DEX, +) medium. (**A**-**B**) RT-PCR analysis of *KGA* (**A**) and *GAC* (**B**) in hTERT-MSC differentiated with osteogenic medium in the presence (+) or in the absence (-) of 2 mM Gln. Gene expressions were normalized on *RPL-15* expression. Data are expressed as fold changes relative to undifferentiated cells maintained at 2 mM Gln =1. Means ± SD of three independent experiments are shown. ***P*<.01 as assessed with one sample t test. # *P*<.05, as assessed with a two-tail Student's t test for unpaired data. (**C**-**E**) RT-PCR analysis of *ALPL* (**C**), and *COL1A1* (**D**) and *SPARC* (**E**) in hTERT-MSC differentiated with osteogenic medium in the presence (+) or in the absence (-) of 1 μM CB-839. Gene expression was normalized on *GAPDH* expression. Data are expressed as fold changes relative to undifferentiated cells =1. Means ± SD of three independent experiments are shown. ***P*<.01, ***P<.001 as assessed with one sample t test. #P<0.5, ## *P*<.01, as assessed with a two-tail Student's t test for unpaired data.

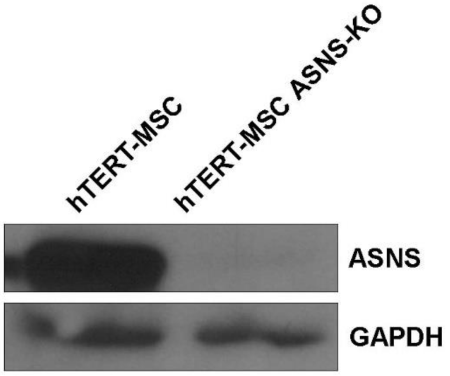

Figure S4. ASNS KO in hTERT-MSC. Western Blot of ASNS in hTERT-MSC and in hTERT-MSC ASNS-KO (as described in Methods) incubated in standard medium. GAPDH was used as loading control. A representative experiment is shown.

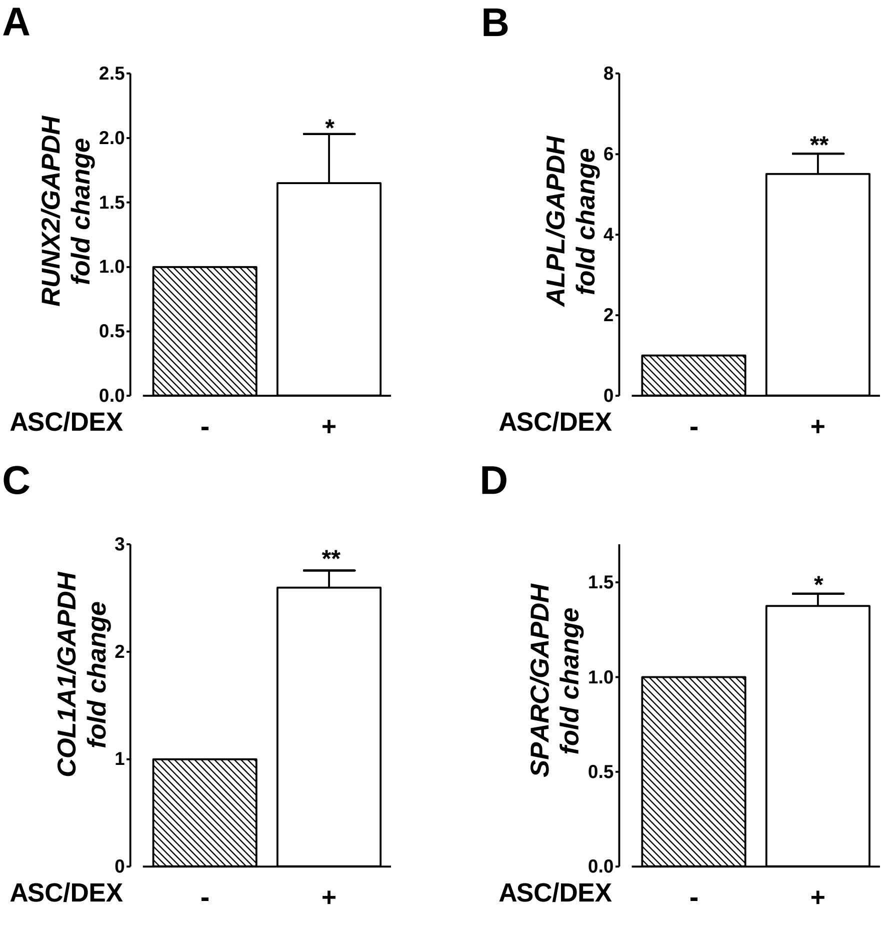

Figure S5. Expression of OB markers during the differentiation of primary bone marrow MSCs. (**A**-**D**) RT-PCR analysis of *RUNX2* (**A**), *ALPL* (**B**), *COL1A1* (**C**) and *SPARC* (**D**) in primary MSC incubated for 14 days in standard (ASC/DEX, -) or osteogenic (ASC/DEX, +) medium. Gene expressions were normalized on *GAPDH* expression. Data are expressed as fold changes relative to undifferentiated cells maintained at 2 mM Gln =1. Means ± SD of experiments performed with MSCs from different donors (n = 3) are shown. ***P<.001 as assessed with one-sample t test.

Original images

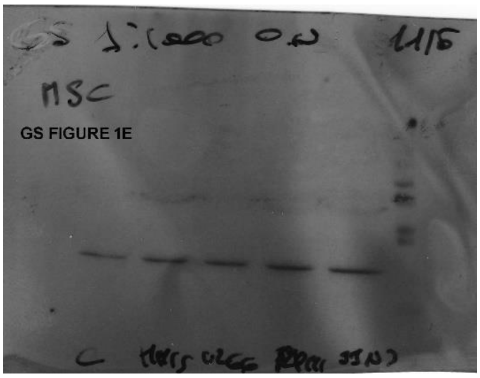

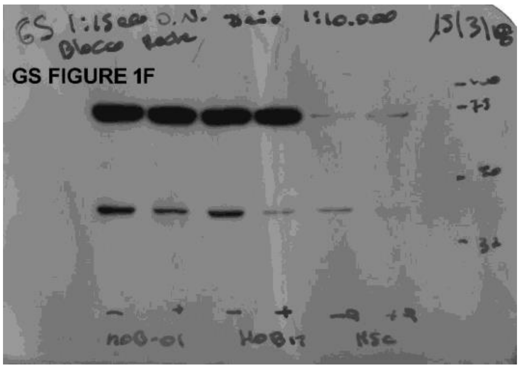

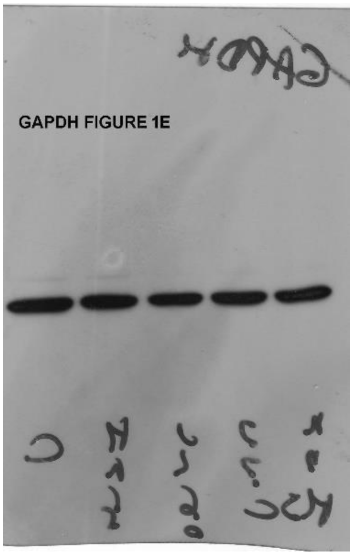

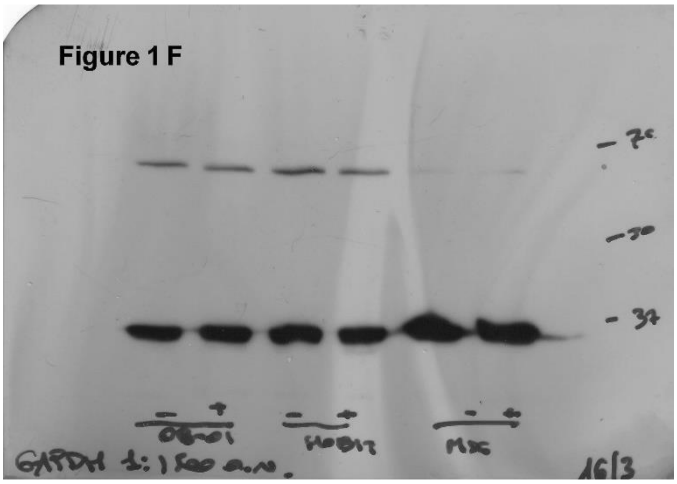

GS FIGURE 2G

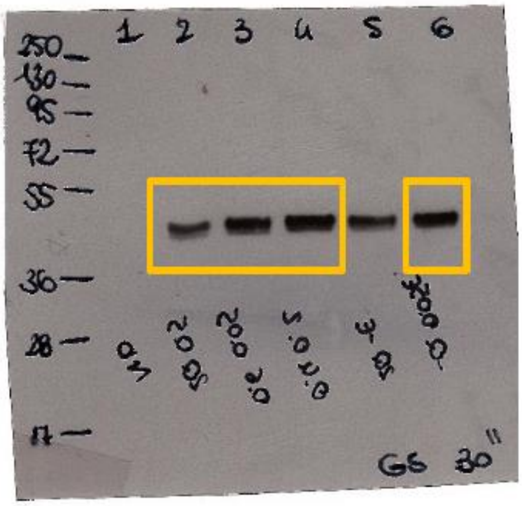

GAPDH FIGURE 2G

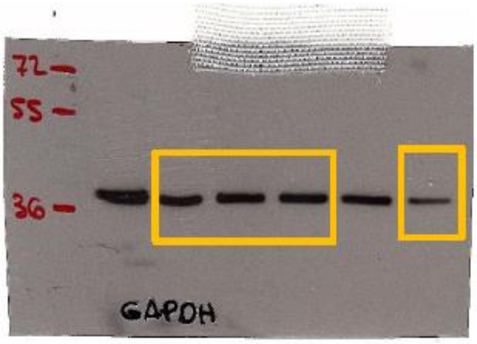

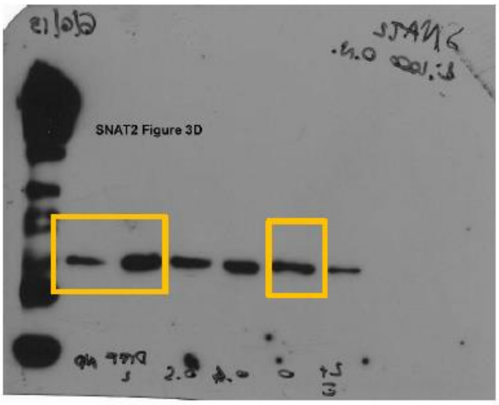
SNAT2 Figure 3D

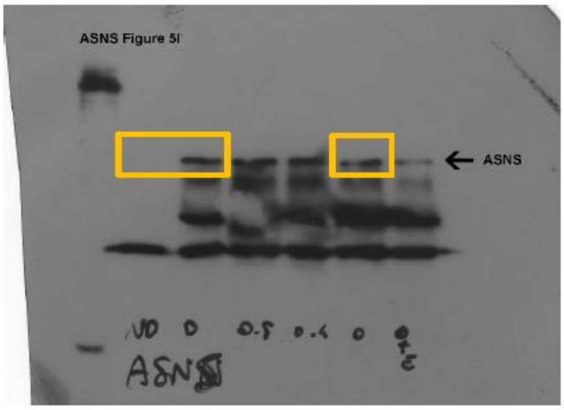
ASNS Figure 5I
ASNS

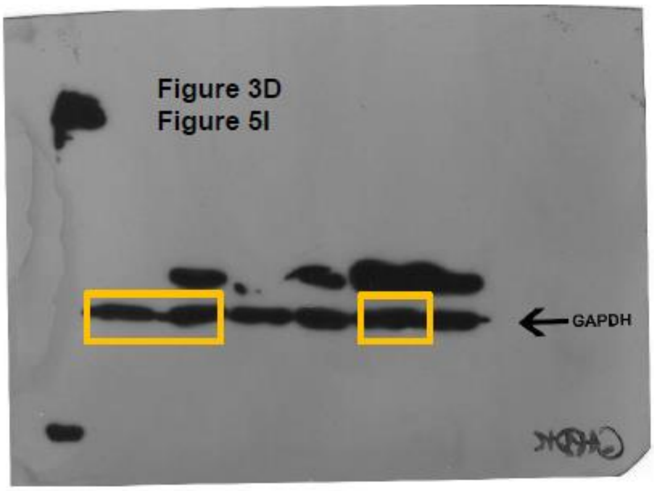
Figure 3D
Figure 5I
GAPDH
